# Bubble velocimetry using the conventional and CNN-based optical flow algorithms

**DOI:** 10.1038/s41598-022-16145-y

**Published:** 2022-07-13

**Authors:** Daehyun Choi, Hyunseok Kim, Hyungmin Park

**Affiliations:** 1 Department of Mechanical Engineering, Seoul National University, Seoul, 08826 South Korea; 2 Institute of Advanced Machines and Design, Seoul National University, Seoul, 08826 South Korea; 3 Present Address: Environment & Safety Research Center, Samsung Electronics, Hwaseong, 18448 South Korea

**Keywords:** Mechanical engineering, Fluid dynamics

## Abstract

In the present study, we introduce new bubble velocimetry methods based on the optical flow, which were validated (compared) with the conventional particle tracking velocimetry (PTV) for various gas–liquid two-phase flows. For the optical flow algorithms, the convolutional neural network (CNN)-based models as well as the original schemes like the Lucas-Kanade and Farnebäck methods are considered. In particular, the CNN-based method was re-trained (fine-tuned) using the synthetic bubble images produced by varying the density, diameter, and velocity distribution. While all models accurately measured the unsteady velocities of a single bubble rising with a lateral oscillation, the pre-trained CNN-based method showed the discrepancy in the averaged velocities in both directions for the dilute bubble plume. In terms of the fluctuating velocity components, the fine-tuned CNN-based model produced the closest results to that from PTV, while the conventional optical flow methods under- or over-estimated them owing to the intensity assumption. When the void fraction increases much higher (e.g., over 10%) in the bubble plume, the PTV failed to evaluate the bubble velocities because of the overlapped bubble images and significant bubble deformation, which is clearly overcome by the optical flow bubble velocimetry. This is quite encouraging in experimentally investigating the gas–liquid two-phase flows of a high void fraction. Furthermore, the fine-tuned CNN-based model captures the individual motion of overlapped bubbles most faithfully while saving the computing time, compared to the Farnebäck method.

## Introduction

Interfacial momentum exchange between continuous and dispersed phases is very important in understanding the physics of multiphase flows, in particular, for the gas–liquid two-phase flows, where the gas–liquid interface deforms in a complex manner and the mutual interaction between the phases is correlated^1–10^. Since the interfacial forces (e.g., drag, lift, added mass, and basset history forces) acting on the rising bubbles in a liquid flow^11–13^ and the bubble-induced agitation (pseudo-turbulence) to the liquid flow^5,8,14–19^ are strongly determined by the bubble velocity (motion) relative to the liquid-phase, it is critical to have the detailed information of bubble velocities. To cope with this, the image-based velocimetry techniques (e.g., two-phase particle image velocimetry and shadowgraphy) to obtain the gas-phase statistics have been proposed widely. Recently, Kim and Park^7^ developed a universal and automated bubble detection method based on the Mask R-CNN deep-learning algorithm, which was shown to be very successful in extracting and tracking the bubble shapes in the images obtained from different flow geometries and optical configurations.

**Figure 1:**
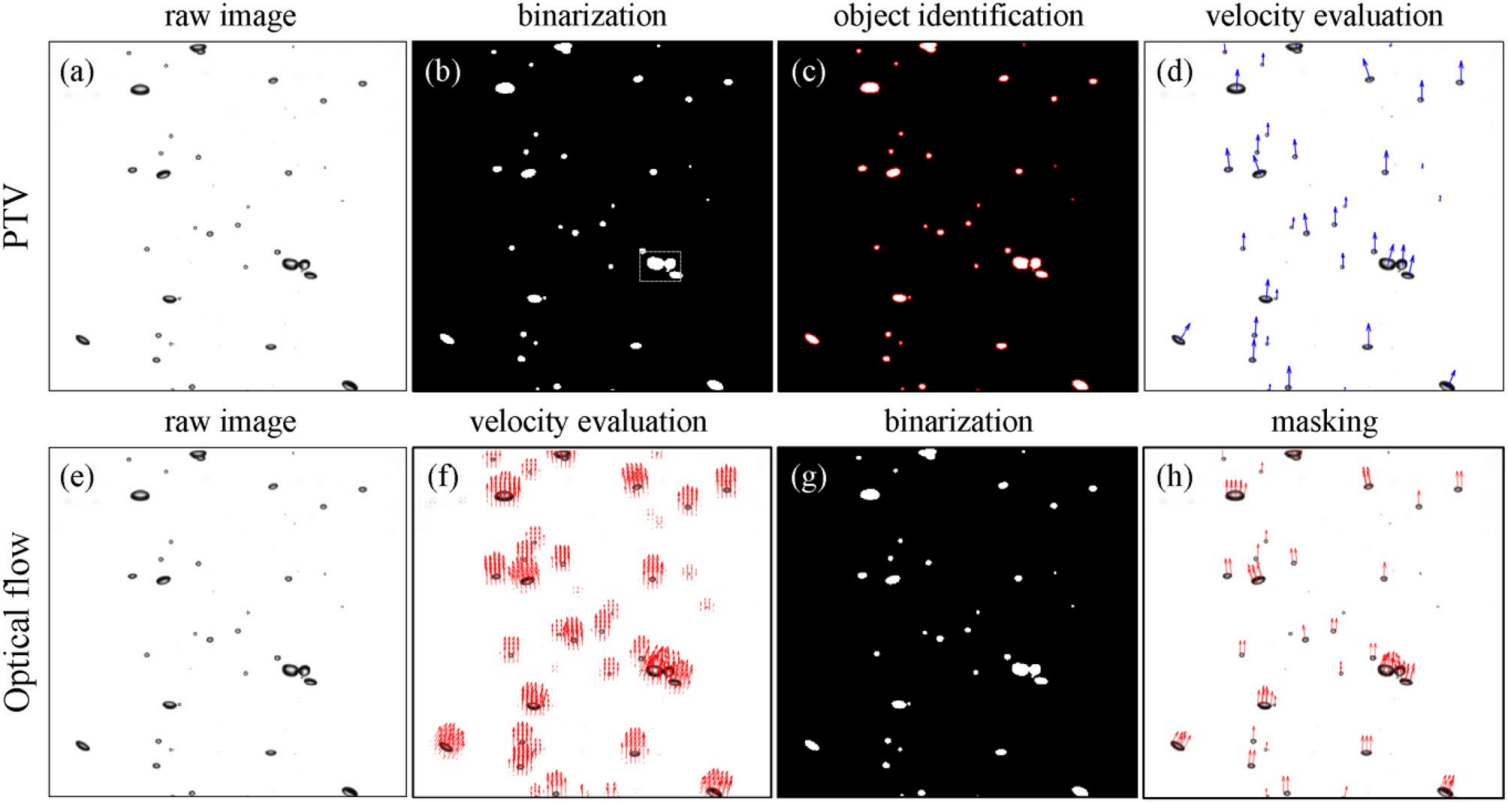
Typical procedure of evaluating the bubble velocities from the shadowgraph image of the bubbly flow: (**a–d**) conventional PTV and (**e–h**) optical flow (Farnebäck) method.

In addition to the bubble shape (i.e., interface morphology), on the other hand, it is also important to measure the bubble velocity, as it is relevant to the interfacial forces. Most of the previous studies have used the particle tracking velocimetry (PTV) algorithm to evaluate the bubble velocity, based on the centroid of each bubble on the images obtained optically. The typical process of PTV applied for the shadow image (taken for the bubbly flow) consists of the binarization, identification, and evaluation, as shown in Fig. 1a–d. First, the bubble is individually recognized and tagged as an identical object in the images that were successively obtained (Fig. 1b,c). Next, the distance traveled by each bubble centroid is divided by the time interval between consecutive images, which results in the bubble velocities (Fig. 1d). The bubble velocity from the PTV is obtained from a clear physical background and thus guarantees the high accuracy, if specific conditions are satisfied such as the low void fraction (< 2.5%) and mild shape deformation, enabling the exact matching of each bubble^4,5,8^. In other words, this conventional method has limitations to be applied to wider circumstances: (i) the computing costs to evaluate the whole velocity fields get increasing significantly with increasing the number of images, (ii) it is difficult to reflect the effect of local deformation of the bubble (only a single velocity vector is obtained for one bubble), and most importantly (iii) it cannot be used for the bubbly flows with a higher void fraction, i.e., for highly overlapped bubbles. The computing cost of the PTV is mainly consumed during the process of identifying individual bubbles. As noted in Table 1, for processing one pair of images, the step of individual object (bubble) identification costs 0.812 s, which is 95% of the entire procedure. On the other hand, the PTV only tracks the bubble centroid to obtain the velocity, and thus, the bubble deformation is ignored, which is known to affect the interaction between the bubble and the surrounding flow^8,20–24^. It would be important as well as interesting to investigate the velocity of the local bubble surface, which is different from that of the centroid^25–27^.

**Table 1:** Comparison of the computing costs of each method for evaluating the bubble velocities in a single pair of dilute plume images (size of 400 × 608 pixels; see Fig. 7).

Cost	PTV	Lucas-Kanade method	Farnebäck method	CNN-based model
Object identification	0.812 s	–	–	–
Vector evaluation	0.041 s	0.025 s	0.155 s	0.033 s
Total	0.853 s	0.025 s	0.155 s	0.033 s

The PTV and the conventional optical flows are operated by CPU (Intel^®^ Core™ i7-5960X CPU @3.00 GHz), whereas the CNN-based optical flow uses GPU (GeForce RTX2080 12 GB). It is noted that only the PTV method requires the step of object (bubble) identification.

**Figure 2:**
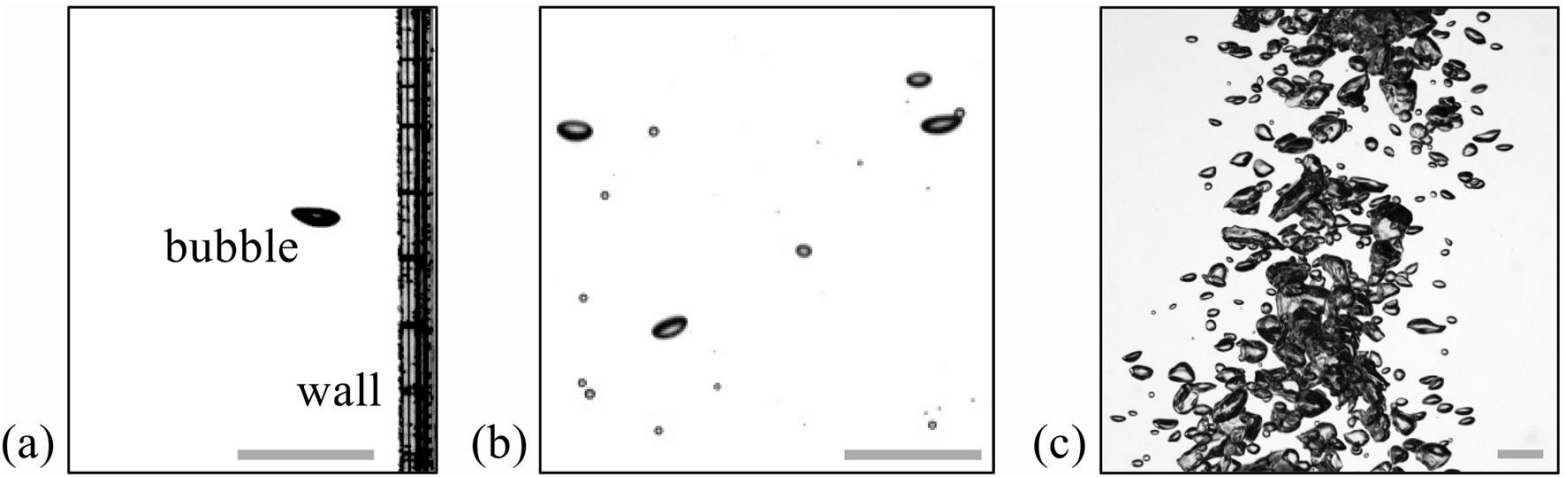
Bubbly flows from different environments: (**a**) single bubble rising near the vertical wall, (**b**) dilute bubble plume (void fraction of 1.13%), (**c**) dense bubble plume (void fraction of 58%). The scale bar at the bottom of each figure denotes 10 mm.

The most critical drawback of the PTV would be the difficulty in calculating the bubble velocity of highly overlapped and deformed bubbles (e.g., see Fig. 2c for the dense bubble plume). Here, the overlap denotes the actual contact of bubbles (or their projections) on the two-dimensional image (see the dashed box in Fig. 1b). Previous studies tried to deal with this issue by dividing the overlapped bubbles using the watershed transform^28^ or ignoring the overlapped bubbles altogether^8^. Watershed transform is a method of recognizing and classifying bubbles based on the bright local maxima that appear at the bubble center region^5^. However, if multiple (more than three) bubbles are simultaneously overlapped or there are multiple maxima in one bubble (typically this happens when the bubble size becomes larger), the bubbles are not clearly distinguished based on the maxima^7^. For the PTV, the identification of the individual bubble is a prerequisite for the velocity evaluation and it can significantly deteriorate the reliability of the calculated velocity vectors. Therefore, the PTV has been mostly used for the case of the relatively small bubble (less than 4 mm of the diameter) experiencing the milder shape deformation, with a lower volume void fraction (Fig. 2a–b and Table 2). However, the bubbly flows that are easily found in nature and industry have a higher volume void fraction, which requires a new method that can accurately and systematically measure the velocity field from a number of deformable and overlapped bubbles.

**Table 2:** Comparison of the tested conditions for measuring the bubble velocities in the bubbly flow using the PIV and/or PTV.

Literatures	Method	Flow type	Bubble shape	Bubble diameter	Local void fraction
Cheng et al.^43^	PIV	Bubble plume	Spherical–ellipsoidal	1.0–2.5 mm	< 30%
Ryu et al.^40^	PIV	Plunging wave	–	~ 3 mm	~ 4%
Seol et al.^41^	PIV + PTV	Bubble plume	Spherical–ellipsoidal	1.5–2.0 mm	0.2–1.8%
Chung et al.^44^	PIV + PTV	Stirred vessel	Spherical	~ 0.5 mm	< 5.4%
Teodori et al.^45^	PIV	Pool boiling	Spherical	1.0–1.3 mm	–
Murgan et al.^42^	PIV + LIF	Bubble plume	Spherical–ellipsoidal	1.1–6.1 mm	–
Cerqueira et al.^46^	PIV + PTV	Bubbly pipe flow	Spherical–ellipsoidal	1.5–4.1 mm	0.7–11.4%
Watamura et al.^47^	PIV + PTV	Micro bubbles	Spherical	~ 0.01 mm	< 0.11%
Present study	Optical flow	Bubble plume	Spherical–irregular	0.1–20 mm	< 58%

As a promising solution to this problem, we propose the optical flow algorithm as a bubble velocimetry method. The optical flow is a technique to evaluate the velocity field based on the change of the light intensity (not dependent on the object) in the consecutive images with the assumptions that (i) the brightness of each material point has a constant value in the consecutive images, i.e., brightness constancy, (ii) the displacement is sufficiently small, and (iii) the flow field is smoothly evolving^29–31^. Here, we brief the general procedure to obtain the vector field using the optical flow algorithm, and the details can be found in elsewhere^32,33^. If the gray value *I*(*x, y, t*) on the position of (*x*, *y*) at any time *t* moves to (*x* + *δx*, *y* + *δy*) after the time interval of *δt*, the brightness constancy condition can be expressed as the Eq. (1).

1 $$I\left(x+\delta x,y+\delta y,t+\delta t\right)=I\left(x,y,t\right)$$

Applying the first-order Taylor series expansion to the left-hand side of the Eq. (1), we have

2 $$I\left(x+\delta x,y+\delta y,t+\delta t\right)\cong I\left(x,y,t\right)+\frac{\partial I}{\partial x}\delta x+\frac{\partial I}{\partial y}\delta y+\frac{\partial I}{\partial t}\delta t.$$

When the Eq. (2) is replaced into the Eq. (1), it is further simplified to ${I}_{x}u+{I}_{y}v=-{I}_{t}$, where ${I}_{x}=\partial I/\partial x, {I}_{y}=\partial I/\partial y$, ${I}_{t}=\partial I/\partial t$, and (*u*, *v*) denotes the velocity field on the (*x*, *y*) plane. In order to solve this equation, the information about two or more points (*x*, *y*), i.e., the gradients, is required, and it is applied on the window basis, instead of a single point. Let’s consider a window of *N* × *M* size (i.e., it consists of points of ***q***_**1**_, ***q***_**2**_, … ***q***_**N**×**M**_). If each point on the window has the same velocity (*u*, *v*), then the Eq. (3) can be established.

3 $$\left[\begin{array}{cc}\begin{array}{c}{I}_{x}({{\varvec{q}}}_{1})\\ \begin{array}{c}{I}_{x}({{\varvec{q}}}_{2})\\ \vdots \end{array}\end{array}& \begin{array}{c}{I}_{y}({{\varvec{q}}}_{1})\\ \begin{array}{c}{I}_{y}({{\varvec{q}}}_{2})\\ \vdots \end{array}\end{array}\\ {I}_{x}({{\varvec{q}}}_{{\varvec{N}}\times {\varvec{M}}})& {I}_{y}({{\varvec{q}}}_{{\varvec{N}}\times {\varvec{M}}})\end{array}\right]\left[\begin{array}{cc}u& v\end{array}\right]=-\left[\begin{array}{c}{I}_{t}({{\varvec{q}}}_{1})\\ \begin{array}{c}{I}_{t}({{\varvec{q}}}_{2})\\ \begin{array}{c}\vdots \\ {I}_{t}({{\varvec{q}}}_{{\varvec{N}}\times {\varvec{M}}})\end{array}\end{array}\end{array}\right]$$

Here, we have *N* × *M* equations for two unknowns, and thus the single solution (*u,v*) can be determined through the least-square method. As noted, the key element of the Lucas-Kanade algorithm is that it approximates the image intensity using the first-order Taylor series expansion^32^. On the other hand, Farnebäck^33^ modified the algorithm such that it can measure the displacement of each point in the consecutive images by assuming that the image intensity is a quadratic function with respect to the position (*x*, *y*). This method costs quite more than the Lucas-Kanade model (see Table 1), but it was shown to provide more accurate and detailed velocity fields.

Recently, the convolutional neural network (CNN)-based optical flow has been suggested to elevate not only the spatial resolution but also the accuracy, compared with the conventional optical flow^34,35^. For example, the series of convolutional networks (FlowNet) with converging and diverging structures have been proven to produce more accurate velocity fields of the single-phase flow with a higher resolution than the conventional optical flow^34^. On the other hand, Sun et al.^35^ proposed the system of the convolutional network (PWC-Net), analogous to the coarse-to-fine adaptive approach of the conventional optical flow (which obtains the velocity fields of the higher spatial-resolution using the pre-calculated velocity field from the under-sampled image), and used it to obtain the velocity field of daily objects (e.g., SINTEL animation, the KITTI dataset, and flyingchairs dataset). It was shown that it is cost-effective and more accurate than the FlowNet and conventional optical flow models. In the present study, we selected this PWC-Net to evaluate the bubble velocity and also re-trained it with the synthetic bubble images.

The network architecture of PWC-Net comprises two fixed-parameter layers consisting of the warping and cost volume, and three trainable-parameter layers consisting of the feature extraction, velocity field estimator, and context network. First, the two consecutive raw images are inserted into the feature extracting layer that is the converging convolutional networks with *n*-levels, and each level of the layer produces a different resolution of ‘features’ (i.e., the product of each convolutional filter). At the lowest-resolution feature, the cost-volume layer and the velocity field estimator evaluate the draft of the velocity field, which is finally converted to the velocity field data through the context layer. This coarser version of velocity data is subsequently updated to the next level of layer and is used to deform one of two features to achieve the better prediction of the velocity field. Likewise, the two features from the consecutive images at the next level of the layer are converted to the velocity vector with a higher resolution. The number of layers for each network and their architecture are explained in the “Method” section. The weights are pre-trained with the KITTI and 3D-FlyingChair datasheet (for the detailed procedure, please refer to Sun et al.^35^), since the application of the CNN-based optical flow has been mainly focused on the identification of large objects such as humans in the avenue, vehicles, and daily objects. However, it has been reported that the CNN-based model can perform like the particle image velocimetry (PIV) and significantly enhances the spatial resolution by fine-tuning (i.e., further training with the dataset of interest)^36,37^, compared to the conventional PIV^38,39^. As an advantage of the optical flow method, they pointed out that it can account for the non-linear deformation of the flow, and claimed that the CNN-based optical flow is capable of measuring the velocity field based on the particle distribution. We are interested in investigating how this CNN-based model would perform in measuring the velocity of highly deformable bubbles, which is more complicated than the translational particle movement.

Previously, the PIV-PTV hybrid algorithm was also proposed as an alternative tool to overcome the limitation of PTV^40–42^. That is, the PIV and PTV were selectively applied in dense and sparse bubble region, respectively, to obtain the bubble velocity field. However, there are two issues in this approach. First, the premise that target particle (or bubble) is drifted by a continuum must precede for the PIV, meaning that two closest particles (which faithfully follow the background fluid) should not intersect to each other and satisfy a continuity, i.e., particle velocities in the interrogation window should be comparable to each other when the window is smaller than the smallest eddy. However, being different from tracing particles, bubble has a slip velocity against the background flow, thus violating the continuity condition. Bubbles often meet or cross each other within the interrogation window, showing the discontinuous velocity distribution (see Fig. 9a). If the PIV is applied to the consecutive bubble-image pair, thus, the inherent dynamics of bubbles (i.e., independent directions and speeds of bubbles) in the same window will be ignored and smoothed, which is not physically acceptable. Secondly, the PIV-PTV hybrid algorithm requires the designation of the threshold to determine which technique to be applied to calculate the velocity of each bubble, which depends on the type of bubbly flow and the optical configuration. Despite this limitation, many previous studies have successfully obtained the bubble velocity using the PIV or PTV(or LIF)-PIV hybrid algorithm for a range of flow conditions such as the flow type, bubble shape, diameter, and local void fraction, as summarized in Table 2. For dense bubbly flows such as the bubble plume^41–43^, stirred vessel^44^, pool boiling^45^, bubbly pipe flow^46^, plunging wave^40^, and ascending microbubbles near the vertical wall^47^, it is found that the bubble size and void fraction considered in the previous studies using the PIV or PTV(or LIF)-PIV hybrid algorithm is less than 10 mm and 30%, respectively. Regarding this, Chung et al.^44^ mentioned that the significant overlapping of the bubble for the higher void fraction (> 30%) greatly reduces the correlation peak of the PIV and ruptures the spatial continuity, which forces the PIV to produce non-physical vectors. Also, it has been reported that the PTV (also PIV) has a difficulty in matching the highly distorted bubbles of larger Weber number^44,46^. On the other hand, the present method is capable of computing the bubble velocity for the void fraction as high as 58% and the bubble size as large as 20 mm, at which the bubbles are irregular-shaped and densely overlapped, which will be discussed later.

Considering the characteristics of above-mentioned approaches, therefore, in the present study, we consider the Lucas-Kanade and Farnebäck models as representatives of the conventional optical flow algorithm, two CNN-based models (PWC-Net) that are pre-trained and fine-tuned, and their applications to some bubbly flows (the rise of a single bubble, dilute bubble plume, and dense bubble plume; Fig. 2) are compared with the results from the PTV. The synthetic bubble images were separately created for the fine-tuning (training), which has been proven to significantly enhance the accuracy of the model^7^. For the validation, the experiments were performed to generate the bubble shadow images in different environments using a high-speed camera. Each optical flow algorithm, together with the PTV, is assessed in terms of the accuracy and time cost. We hope the present results will be widely utilized in relieving the difficulties in the dynamic analysis of the optical images having multiple objects, and accelerating the experimental studies of bubbly flows in complex geometries.

## Configuration of the optical flow model

### Conventional optical flow model

**Figure 3:**
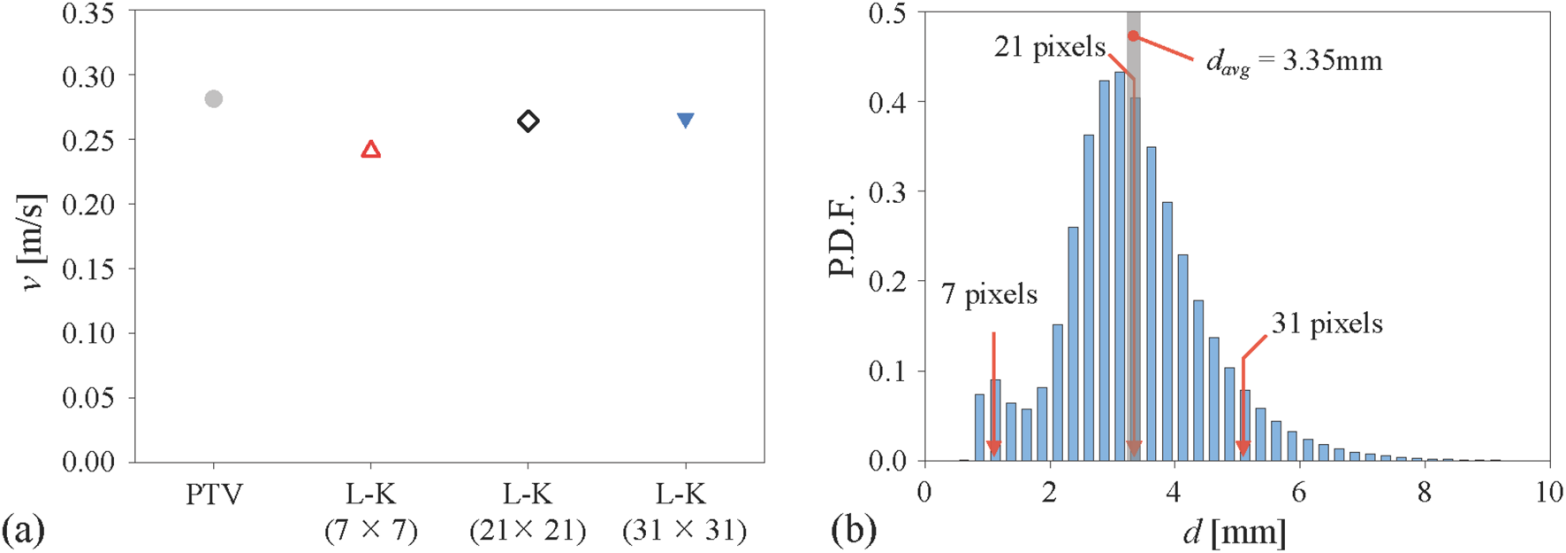
Effect of window size on the optical flow measurement: (**a**) averaged vertical velocity (filled circle, PTV; open triangle, Lukas-Kanade method with the window size of 7 × 7; open diamond, 21 × 21; inverted filled triangle, 31 × 31 pixels); (**b**) probability density function of measured bubble size (solid lines denote the tested window sizes). Here, the measurements were done for the dilute bubble plume (Fig. 2b) with the mean bubble diameter of 3.4 mm and void fraction of 1.13%.

For the conventional optical flow, two consecutive bubble images are segmented into a small window, in which the displacement vectors are generated. Therefore, the density of the window determined by its size and overlap ratio is related to the spatial resolution of the velocity field, and thus, they should be carefully selected. To examine the dependency on the window size, the vertical velocity of the bubbles in a dilute plume, shown in Fig. 2b, is measured with three different window sizes of 7 × 7, 21 × 21, and 31 × 31 pixels using the Lukas-Kanade method. Figure 3a shows that the vertical velocity tends to be underestimated as the window size becomes smaller, compared to the result of the PTV. This indicates that there exists a certain threshold of window size above which the optical flow would perform well, which is found to depend on the bubble size. The probability density function of the measured bubble size distribution is plotted in Fig. 3b, together with the relative position of three window sizes. As shown, the window size of 7 × 7 pixels is smaller than the averaged bubble size of 3.4 mm, but two larger windows are comparable (or larger) than that. Thus, it is learned that the window size should be at least as large as the average size of the bubbles that are to be measured. Based on this finding, we tested various sizes of windows for Lukas-Kanade and Farnebäck methods, by applying them to the flows with lower number density of bubbles (Fig. 2a,b). For the Lukas-Kanade method, as we have discussed, the accuracy of the measured bubble velocity was enhanced as the window size is slightly larger than the averaged bubble diameter, while the Farnebäck method was less sensitive to the window size. As a result, the window size for the Lukas-Kanade method was set to 21 × 21 pixels for the single bubble (Fig. 2a) and 31 × 31 pixels for bubble plumes (Fig. 2b,c). It was fixed as 7 × 7 pixels for the Farnebäck method. The overlap ratio of 50% and 0% was applied for the Lukas-Kanade and Farnebäck methods, respectively. Since the conventional optical flow evaluates the velocity vector by the window, velocity fields cover a slightly larger area than the bubbles (Fig. 1f), and a simple masking process is applied afterward to remove the velocity vectors located outside of the bubble. Here, the binarization process is the same as that of the PTV (Fig. 1b). Since the optical flow technique does not require the object identification (Fig. 1c), it costs much less time (see Table 1).

### CNN-based optical flow model

**Figure 4:**
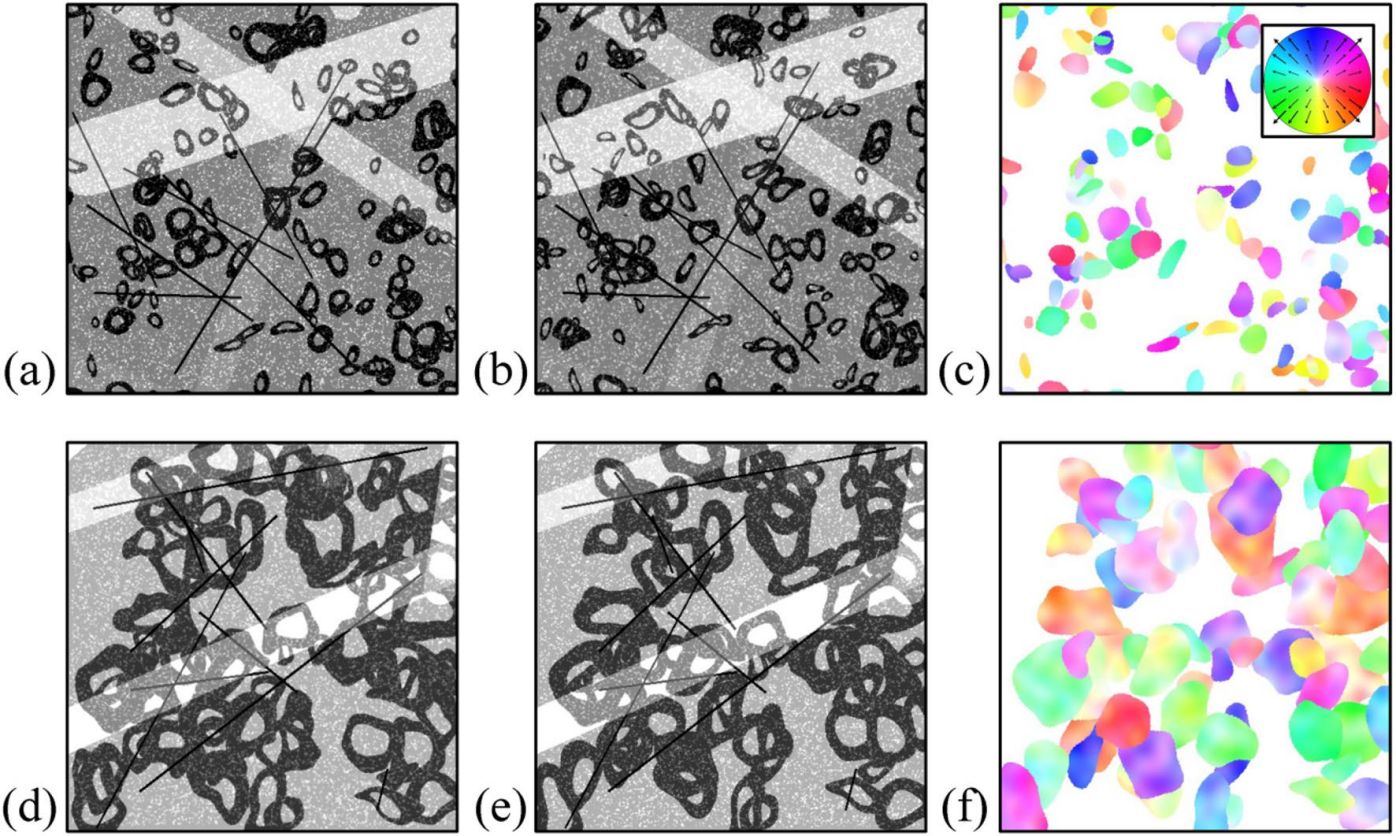
Example of synthetic bubble image pairs used for the fine-tuning dataset for the PWC-Net: (**a,b**) small bubbles (bubble size = 10–60 pixels); (**d,e**) large bubbles (bubble size = 40–130 pixels); (**c,f**) contour of bubble velocity corresponding to each pair. The inset figure in (**c**) shows the direction and magnitude of velocity, expressed in terms of the hue and saturation, respectively.

In contrast to the conventional optical flow, the PWC-Net^35^ is free from the window size because the whole velocity vector field is obtained at once. Instead, the hyperparameter (e.g., number of layers, epochs, and batch size) for the networks may affect the estimation. The model is trained following the learning schedule, as introduced in Sun et al.^35^, which reduces the learning rate (initially starting from 10^–5^) by half at 160 k, 240 k, 320 k, and 400 k epochs, with the batch size of 4. As the trainable layers, the convolution filters of feature extraction, context section, and velocity field estimator and their network are used, of which the details are described in the Method section. To examine the effect of such parameters, we used the weights of the model both pre-trained by the previous work^35^ and fine-tuned with the synthetic bubble images and their velocity fields, generated by the in-house Python code. While developing the deep learning-based algorithm, it is regarded that the generation of proper set of synthetic (or natural) data for training and validation is important as much as the development of the architecture of the neural network, especially, when the labeled dataset is not sufficiently available ^7,48^. To the best of our knowledge, the dataset (the bubble image and its velocity field) suitable for the present study does not exist, indicating that even if there are good architectures for the optical flow model, it is not feasible to be applied as a bubble velocimetry. Therefore, it is required to generate the bubble-image-velocity dataset that enables the model to operate in the real bubble images. By delicately synthesizing and evenly distributing the dataset, the neural network can also avoid the biased prediction and increase the robustness to the corner case^48^. Figure 4 shows the representative synthetic bubble images and the corresponding velocity contours. The shape of the synthetic bubble was designated to be a randomly deformed ellipse with the size of 10–130 pixels, including the local maxima (i.e., the bright area inside the bubble shadow image caused by the refraction of background light) at its center region (Fig. 4a,d). The number of bubbles is 50–200 in one image pair. The synthetic bubbles are displaced with the random displacement of 10–50 pixels per frame (Fig. 4b,e), by which the corresponding velocity fields can be obtained as shown in Fig. 4c,f, respectively. To enhance the robustness to the noises that can exist in bubble images, the intensity of the background was varied in the range of 0.1–0.9 (0 and 1 stands for the darkest and brightest intensity, respectively) at each pair, and the transparent rectangular bars, thin solid lines, and particles with the size of 1–5 pixels are also positioned at random, to represent the possible noise that can be intervened in the optical imaging. Finally, 100,000 pairs of bubble images (and the velocity fields) were generated and used to fine-tune the weights of the networks. The optimizer is selected as the ADAM scheme. The specification of the workstation that we have used is a single NVIDIA RX 2080 Ti GPU, and the calculation procedure (velocity field is calculated from the network and masked based on the binarization) is similar to the conventional optical flows (Fig. 1e–h).

### Comparison on the computational costs

The computational cost of each method is summarized in Table 1. Processing a single pair of images taken for the dilute plume (Fig. 2b) to obtain the bubble velocities took 0.85, 0.025, 0.16, and 0.033 s for the PTV, Lukas-Kanade, Farnebäck, and CNN-based models, respectively. Here, the CNN-based model used the GPU (GeForce RTX2080 12 GB) to calculate the vector field, whereas the other models used the CPU (Intel^®^ Core™ i7-5960X CPU @3.00 GHz). As explained, the PTV method requires the longest computational time, mainly originating from the bubble identification step. The Lukas-Kanade takes the smaller cost than the CNN-based model, thanks to its simple assumption on treating the image intensity. On the other hand, the cost of the Farnebäck method is much greater than the CNN-based model, due to the higher-order modeling. Together with this comparison, we will discuss the reliability (accuracy) of the measured bubble velocities in the below. Here, the computational cost based on the sampling time (or the total amount of processed bubbles) is not compared, because such comparison would be unfair in accordance with the acquisition scheme. The sampling time (*t*_*s*_) required for the converged statistics will increase with decreasing time interval (*dt*) between two paired images, when the bubble images are measured at regular time intervals (say, for *dt*). Since, in general, the time interval (*dt*) for the conventional optical flow method is shorter than that of the PTV, thus, the required number of frames and the time cost will be larger for the conventional optical flow method, given the same sampling time. However, this kind of simple acquisition scheme is avoided when using the high-speed imaging for the sake of converged statistics. For example, the double-shutter acquisition enables the long sampling time by distancing the pairs of bubble image while shortening the time interval (*dt*) between two images, which is commonly performed in measuring the turbulent bubbly flow^8^. Through this, the sampling time can be decoupled with the time interval (*dt*) between consecutive images, which could be realized to be sufficiently small for the specific algorithm such as the conventional optical flow methods. Therefore, the most relevant source of the computing cost will be fairly judged by the processing cost consumed for single pair of bubble images.

## Results and discussion

### Evaluation of the optical flows as a bubble velocimetry

To compare the characteristics and performance of optical flow methods, each algorithm is applied to the bubbly flows of different conditions (Fig. 2). First, we will start with the simplest case of a single bubble rising near the vertical wall, with the periodic lateral oscillation (Fig. 2a), and next discuss the results with the rise of several bubbles (without a significant overlap) in a dilute plume (Fig. 2b). Finally, the case of large population of deformable bubbles (with a significant overlap) in a dense plume (Fig. 2c) will be tested.

### Single bubble rising near the vertical wall

**Figure 5:**
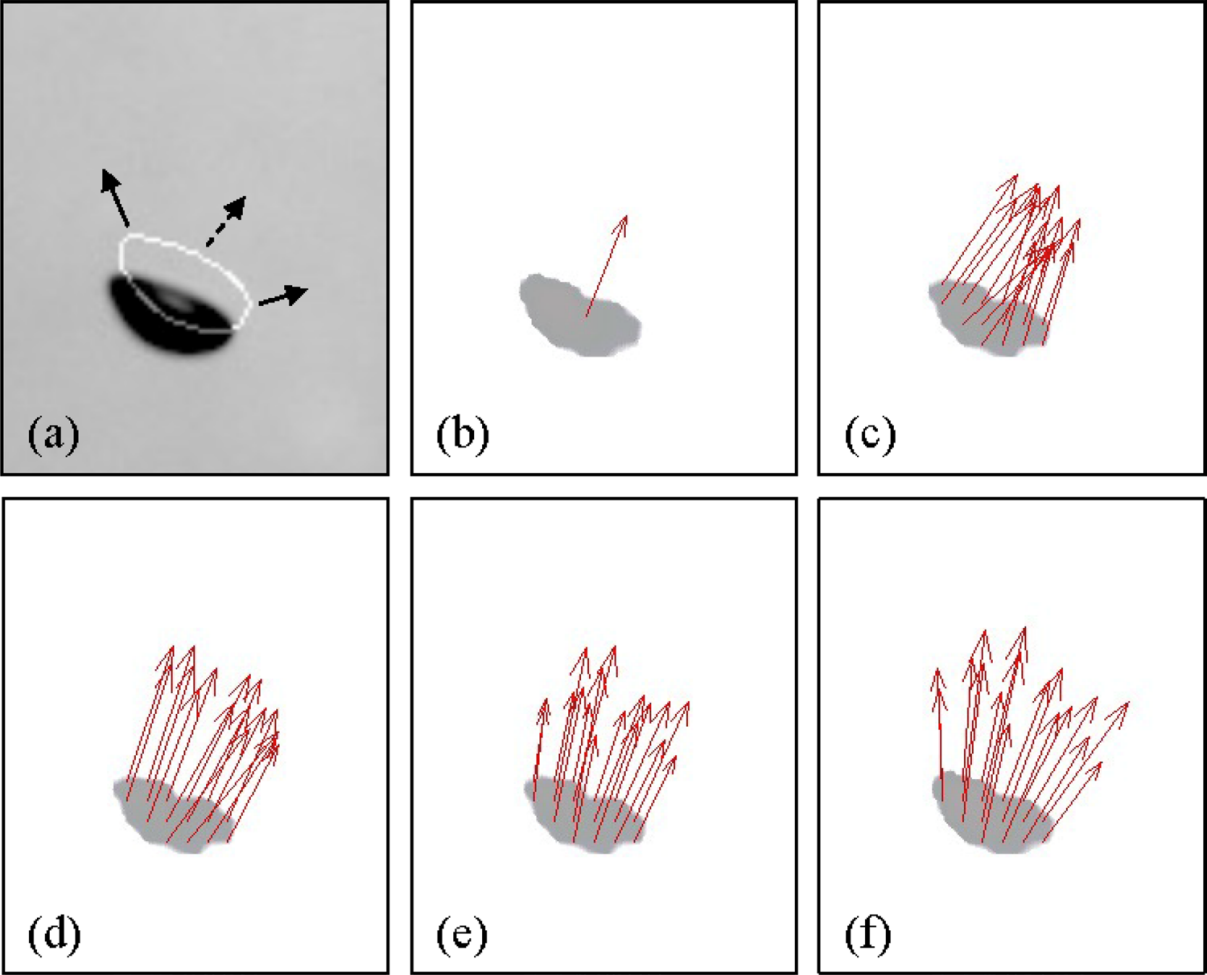
Evaluation of the velocity of a single bubble (size of 3.5 mm): (**a**) the instant raw image with the bubble morphology at the next time step (after 30 ms) overlapped with a solid line; (**b**) result of the PTV; (**c**) Lukas-Kanade method; (**d**) Farnebäck method; (**e**) pre-trained CNN-based model; (**f**) fine-tuned CNN-based model. The gray shadow in (**b–f**) corresponds to the bubble shadow in (**a**).

Figure 5 compares the velocity vector of a single bubble near the vertical wall, obtained by each method. As noted, one velocity vector at the bubble centroid is captured from the PTV (Fig. 5b), while the optical flow algorithms provide multiple velocity vectors, i.e., including the information about the local movement (deformation), assisted by the intensity-based measurement mechanism (Fig. 5c–f). Regardless of the method used, it is observed that the velocity vectors are oriented along the same direction, while the detailed pattern of velocity distribution differs. With the Lucas-Kanade method, velocity vectors located at the periphery of the bubble, characterized by the large curvature, tend to tilt toward the averaged travel direction of the bubble (Fig. 5c), whereas they are directed outward with the Farnebäck and two CNN-based models (Fig. 5d–f). Observing the interfacial deformation of the bubble, the peripheral regions expand while the middle area of the bubble tends to bulge up (see the arrows in Fig. 5a), which is more reliably detected by the Farnebäck and CNN-based models than the Lucas-Kanade method.

**Figure 6:**
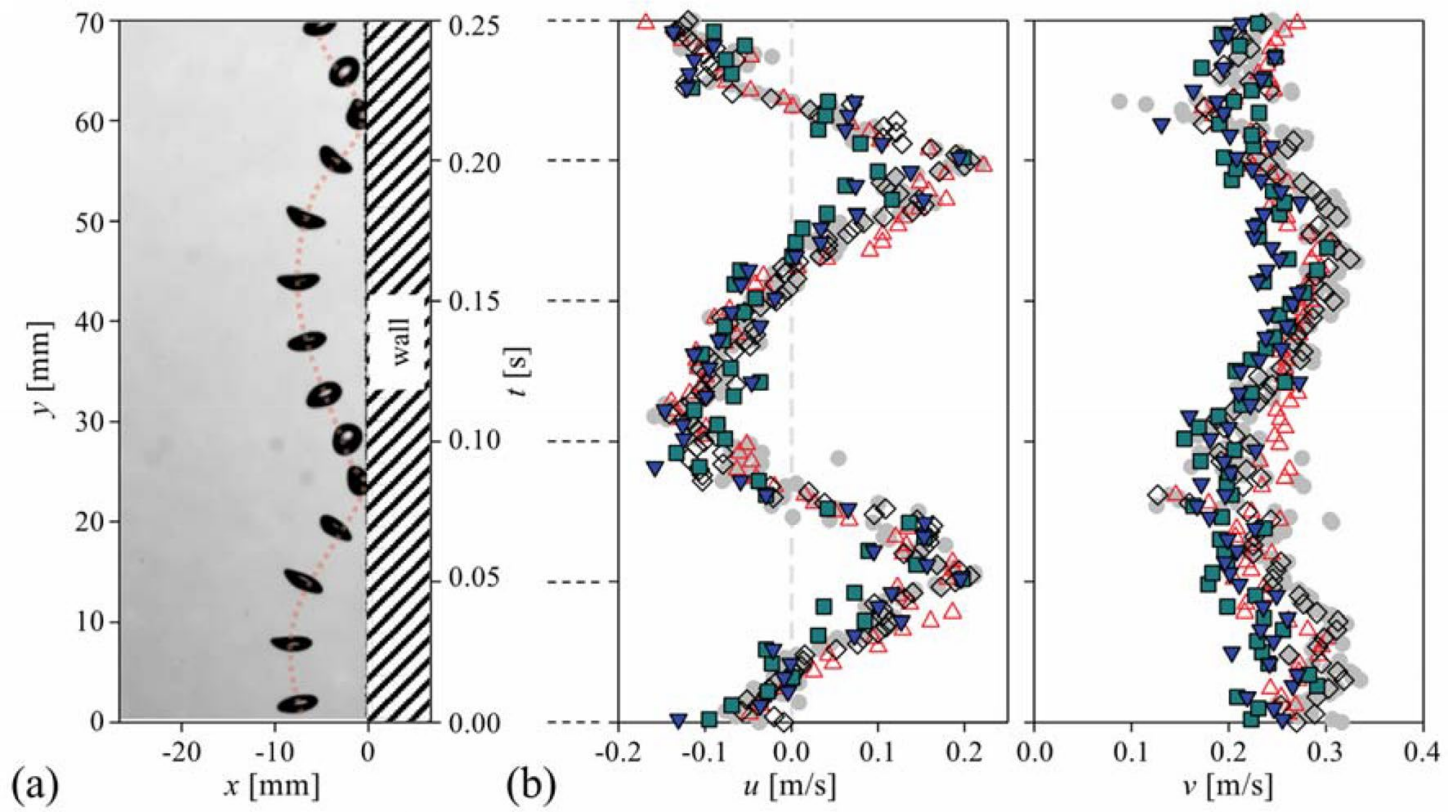
Evaluation of the velocity of a single bubble rising (with bouncing) near the wall: (**a**) sequential bubble shadow images with the time interval of 20 ms. The dotted line denotes the bubble trajectory. (**b**) Corresponding time history of the horizontal (*u*) and vertical (*v*) bubble velocities: filled circle, PTV; open triangle, Lukas-Kanade method; open diamond, Farnebäck method; filled square, pre-trained CNN-based model; inverted filled triangle, fine-tuned CNN-based model.

For further quantitative evaluation, the time history of horizontal and vertical velocity components (for the same bubble in Fig. 5) is plotted in Fig. 6. For the data obtained from the optical flow methods, the averaged value of the velocities inside the bubble is used as a representative velocity. As shown, it is found that optical flow methods can accurately measure the temporally varying velocities of a moving bubble, being compared with the conventional PTV data. It is interesting to see that the pre-trained PWC-Net^35^ can reasonably provide the bubble velocity data suggesting its versatility, although it was not trained (or optimized) for the bubble velocimetry. However, the pre-trained CNN model predicts the slightly lower horizontal velocity at *t* = 0.08 and 0.22 s, which correspond to the instances when the bubble collides with the wall, and it is thought that the pre-trained model is limited in distinguishing the wall and bubble shadow (Fig. 6b). On the other hand, the fine-tuned model (re-trained with the synthetic bubble images) seems to overcome this limitation and predicts the bubble velocities better at the instants of bubble-wall collision. For the vertical bubble velocity, the CNN-based models tend to slightly underestimate, compared to other methods when the bubble deformation is the largest (e.g., at *t* = 0.03 s and 0.18 s in Fig. 6b). At this time, the bubble aspect ratio increases rapidly, i.e., the bubble shape shrinks abruptly along the rising (vertical) direction, by which the larger drag force acts on the bubble decelerating its movement. As noted, each method tested in the present study has a different dependency (or sensitivity) on the local bubble deformation (Fig. 5), and the traditional PTV cannot reflect the localized interfacial movement. It is noted that the relatively larger scatter in the data obtained by the CNN-based algorithms is attributed to the enhanced sensitivity to the bubble deformation. This also indicates that it would require additional caution to interpret the velocity vectors obtained by the optical flow methods. Although they were averaged just for the purpose of comparison in Fig. 6, it is clear that the averaged velocity would not represent the actual bubble dynamics. After it is confirmed that the optical flow-based bubble velocimetry is feasible, it will be interesting as a future work to define each velocity and how they can be applied to the complex problems such as the mechanism of wobbling bubble^49–51^.

### Dilute bubble plume (void fraction < 10%)

**Figure 7:**
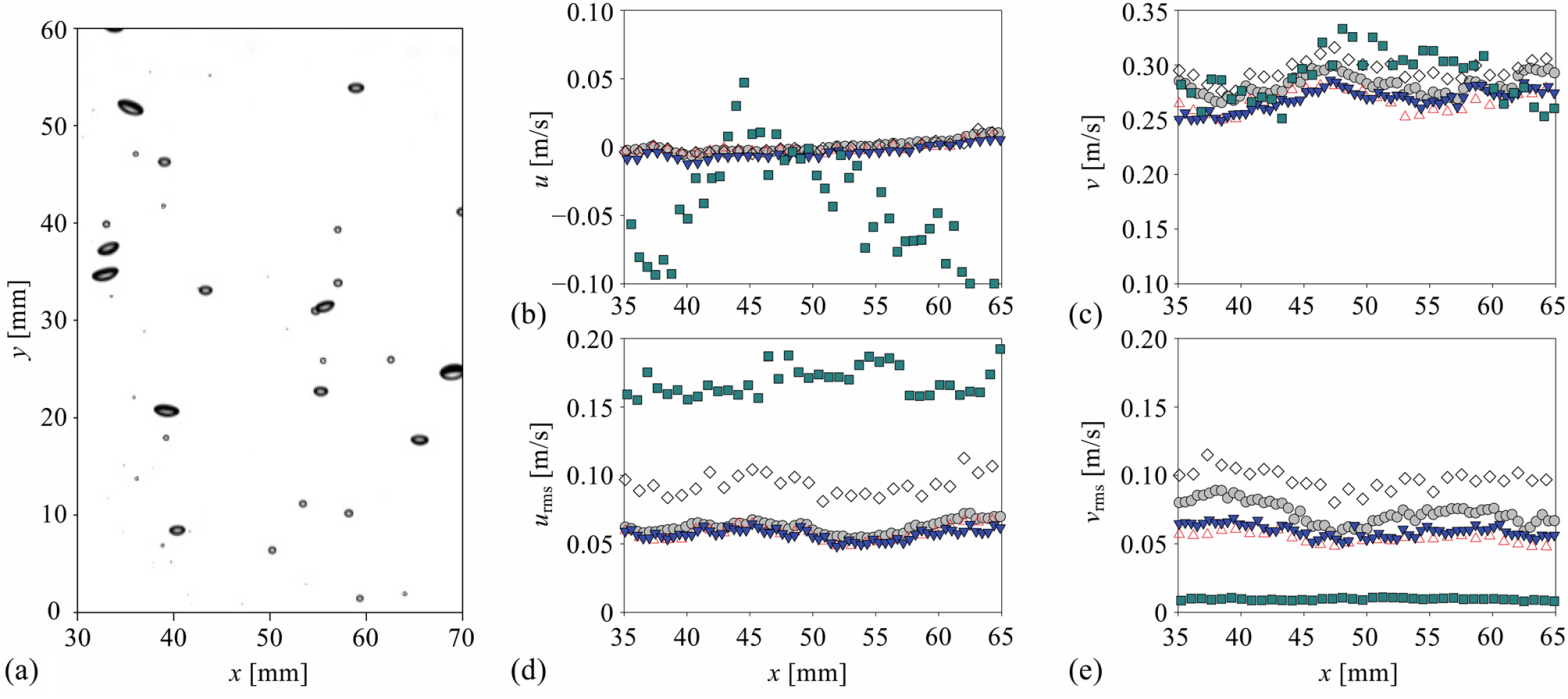
Evaluation of the bubble velocity in the dilute bubble plume (1.13% void fraction): (**a**) instantaneous image of the dilute bubble plume; (**b**) horizontal velocity; (**c**) vertical velocity; (**d**) horizontal root-mean-square (r.m.s.) velocity; (**e**) vertical r.m.s. velocity. All velocity components were averaged in time and *y*-direction. filled circle, PTV; open triangle, Lukas-Kanade; open diamond, Farnebäck; filled square, pre-trained CNN-based model; inverted filled triangle, fine-tuned CNN-based model.

Figure 7 shows the time-averaged and root-mean-squared (r.m.s.) fluctuating velocities for the case of the dilute bubble plume (void fraction of 1.13%). The representative bubble shadow images evaluated are shown in Fig. 7a, and the velocity profiles are obtained by averaging along the vertical (*y*) direction, as well. The time-averaged horizontal and vertical velocities were around 0 and around 0.3 m/s, which agrees with the previous result of the bubble plume with a similar condition (void fraction of 0.5–10%) (Riboux et al.^1^). The conventional optical flow methods and fine-tuned CNN-based model provided quite accurate time-averaged velocities in both directions, compared to the PTV (Fig. 7b,c). However, unlike the case of a single bubble velocimetry, the mean bubble velocities estimated by the pre-trained PWC-Net tend to deviate a lot; in particular, the horizontal velocity did not represent the statistically symmetric nature of the uniform bubbly plume. This discrepancy clearly implies the importance of choosing the proper set of data to train the deep learning model. The pre-trained model (in this case, the original model was trained by the KITTI, SINTEL, and flyingchair dataset (Sun et al.^35^) is optimized for calculating the velocities under the limited conditions (e.g., small void fraction of < 0.2%) since it was trained with the image of objects with number of less than 10. In contrast, the re-trained PWC-Net was shown to measure the velocities of moving multi-objects (~ ***O***(10^2^)), as seen in Fig. 7b,c. In terms of the vertical velocity (*v*), compared to the PTV data, the maximum deviation of the Lucas-Kanade, Farnebäck, pre-trained PWC-Net, and fine-tuned PWC-Net were 9.5, 8.8, 16.8, and 12.4%, and the average errors were 5.4, 4.6, 6.9 and 4.7%, respectively. Thus, it can be said that all the models except for the pre-trained CNN-based one showed a reasonable accuracy for the estimation of the first-order statistics of the bubble velocity.

In terms of the r.m.s. vertical and horizontal velocity fluctuation (Fig. 7d,e), interestingly, the performance of tested models is quite different. Following the mean vertical velocity, the pre-trained PWC-Net showed the most unreliable prediction, i.e., over- and under-estimation of the horizontal and vertical velocity fluctuations, respectively. It was found that the Lucas-Kanade method and the fine-tuned PWC-Net measure the r.m.s. velocity fluctuation quite accurately; the horizontal component agrees with the result of PTV while the vertical velocity fluctuation is slightly lower. This is related to the nature of the optical flows to capture the interfacial velocity, not the velocity of the bubble centroid, as we have discussed in Fig. 6b. With the Farnebäck method, the r.m.s. fluctuation velocities in both directions were estimated to be quite higher compared to the PTV, which is attributed to the wide spatial variation in the calculated velocity, indicating that the assumption of the higher-order distribution of intensity would be over-specified and degrade the bubble velocity measurement. Similarly, the Lukas-Kanade method recognizes the image intensity as a smoothed image, resulting in the underestimation of r.m.s. fluctuation velocity (Fig. 7e). However, the CNN-based models are free from these assumptions and provide the results closest to the PTV.

### Dense bubble plume (void fraction > 10%)

**Figure 8:**
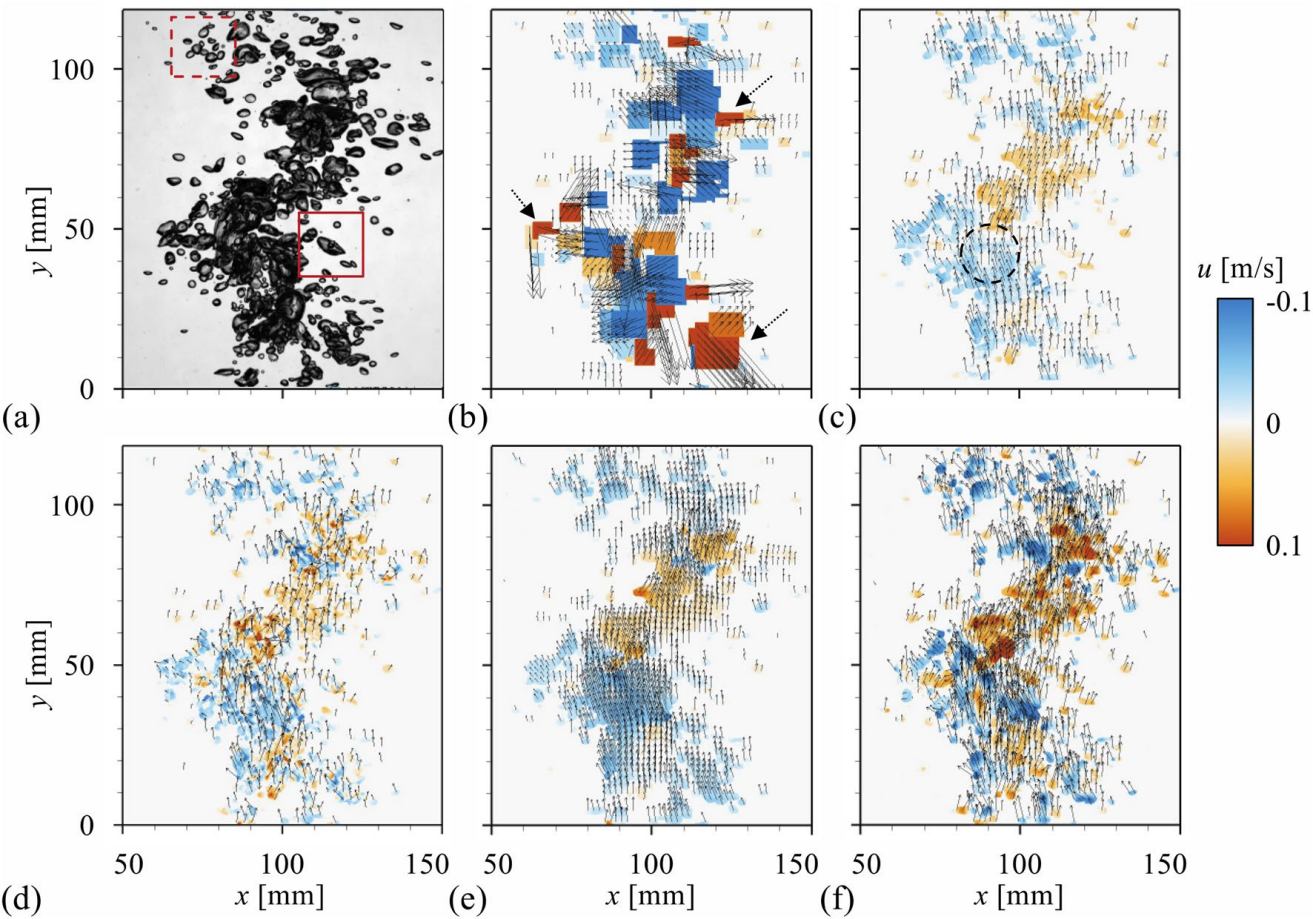
Evaluation of the bubble velocity distribution in the dense bubble plume (58.1% void fraction): (**a**) raw image; (**b**) PTV; (**c**) Lukas-Kanade; (**d**) Farnebäck; (**e**) pre-trained CNN-based model; (**f**) fine-tuned CNN-based model. In (**b–f**), the color contour denotes the magnitude of the horizontal bubble velocity.

Encouraged by the performance of optical flow algorithms as a bubble velocimetry, we applied the methods to the highly dense bubble plume with the void fraction of 58% (Fig. 2c), for which the conventional PTV-based approach is expected to suffer from the highly overlapped bubbles. Thus, the evaluation on the accuracy based on the comparison with the PTV would not be valid, and we will focus on examining how the measured bubble velocity using the optical flows is physically acceptable. In previous studies, the relative bubble velocity was assumed to be constant in such a highly dense bubble plume^22^. Obviously, they did not consider the spatial distribution of bubble size or void fraction, which affects the force acting on the bubbles and determines the interfacial momentum transfer. Naturally, this strong assumption makes only the analysis of flow characteristics on a large scale (say, plume scale) possible. In this background, the bubble velocimetry suggested in the present study would make it possible to quantify the relative bubble velocity in time and space, enabling the more accurate analysis of bubbly flow phenomena in terms of not only the plume scale but the smaller scales. The velocity fields (and the corresponding contour of horizontal bubble velocity) obtained by applying each method to the instantaneous flow (Fig. 8a) are shown in Fig. 8b–f. With the PTV (Fig. 8a), the highly entangled bubbles make it almost impossible to identify the individual bubbles and they are recognized as one big object (represented by the large rectangular contours, of which the shape and size change inconsistently), and the non-physical velocity vectors (indicated by the arrows in Fig. 8b) directing the opposite direction to the bubble movement are obtained. On the other hand, the optical flow methods are capable of revealing the unsteady kinematics and dynamics of the bubble plume as well as the bubble velocity distribution (Fig. 8c–f). Actually, to the best of our knowledge, this kind of quantitative characterization of the bubble velocities in the bubbly flow at the void fraction as high as 50% was not attempted and successful so far. Comparing the results among optical flow models, the details of the spatial variation in the measured velocity fields are found to be different. For the Lucas-Kanade method (Fig. 8c) and pre-trained PWC-Net (Fig. 8e), the spatial distribution of velocity is smoothed out (for example, see the area noted with dashed circle in the figure) at the positions with many overlapped interfaces (Fig. 8a). This is, respectively, attributed to the low-order assumption applied to the image intensity and the lack of relevant training with the multibody-velocity data, which were compensated by the Farnebäck and fine-tuned PWC-Net, respectively. As shown in Fig. 8d,f, their results are relatively successful in detecting the sharp interfacial variations.

**Figure 9:**
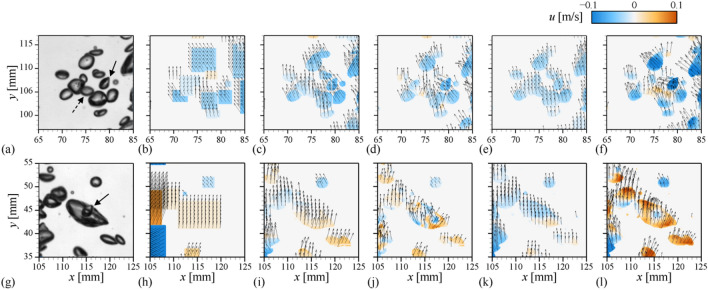
Instantaneous velocity field with the horizontal velocity contour for the local areas highlighted in Fig. 8a: (**a,g**) raw image; (**b,h**) PTV; (**c,i**) Lukas-Kanade; (**d,j**) Farnebäck; (**e,k**) pre-trained CNN-based model; (**f,l**) fine-tuned CNN-based model.

To examine this in detail, the bubble velocity distribution in the areas marked with dashed and solid boxes in Fig. 8a are shown in Fig. 9b–f and h–l, respectively. The tested instantaneous images are again shown in Fig. 9a,g, respectively, which show that the individual bubbles in the bubble cloud move in different directions to each other (see the supplementary movies 1 and 2, respectively). With the PTV (Fig. 9b,h), the overlapped bubbles are again recognized as a large area with the same horizontal velocity, failing to represent the actual bubble movements. The Lucas-Kanade (Fig. 9c,i) and pre-trained PWC-Net (Fig. 9e,k) produce the velocity vectors on each bubble to be directed along the same orientation, i.e., smoothed out artificially, and the horizontal movements of each bubble observed in supplementary movies 1 and 2 cannot be captured. On the contrary, the Farnebäck method (Fig. 9d,j) and fine-tuned PWC-Net (Fig. 9f,l) are capable of faithfully tracking and quantifying the movements of each bubble independently. Surprisingly, it is found that they can measure the velocity of the small bubble partially or fully covered by the larger ones (Fig. 9j,l), which is quite promising to consider that the conventional particle image velocimetry (PIV) is limited in evaluating the velocities across a wide range of scales simultaneously^37,38^ and the similar issue was raised in the recent deep- learning based bubble detection^7^. For example, the bubble indicated by the dashed arrow in Fig. 9a solely translates to the right direction, while the surrounding bubbles are moving left (see the supplementary movie 1). This movement can be detected by the Farnebäck method (Fig. 9d) and fine-tuned PWC-Net (Fig. 9f), evidenced by the contour of horizontal velocity. As another example, the smaller bubble noted by a solid arrow in Fig. 9g) is located inside the larger one and they have opposite horizontal velocities (see the supplementary movie 2). For this case, only the re-trained CNN-based model can accurately identify the velocity of the small bubble (Fig. 9l), while others produced unrealistic results. Thus, it is understood that the well-trained CNN-based optical flow, which is independent of any assumptions on the image intensity and the size effect of interrogation window, is the most robust and reliable on the interfacial complexity of bubbly flow.

**Figure 10:**
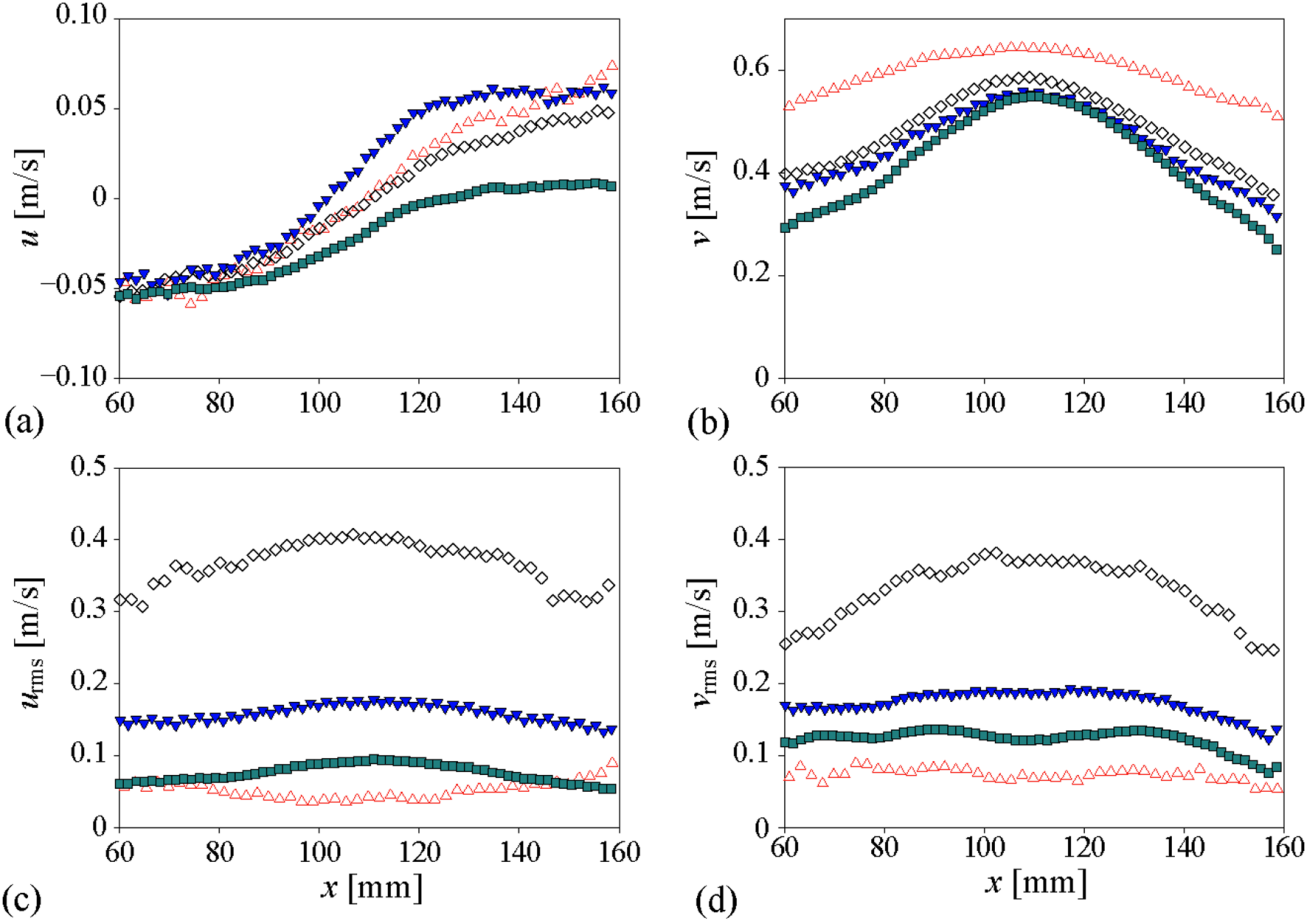
Bubble velocity profiles in the dense bubble plume: (**a**) time-averaged horizontal velocity; (**b**) time-averaged vertical velocity; (**c**) horizontal r.m.s. velocity fluctuation; (**d**) vertical r.m.s. velocity fluctuation. Open triangle, Lukas-Kanade; open diamond, Farnebäck; filled square, pre-trained CNN-based model; inverted filled triangle, fine-tuned CNN-based model. All velocities were averaged along the vertical (*y*) direction in the measurement plane.

Finally, the quantitative comparison of the velocity statistics obtained by each approach is provided in Fig. 10, which were spatially and temporally averaged. For the time average, we measured the bubble plume for 6000 s and averaged all obtained instantaneous flows, consisting of 20,600 image pairs. For the spatial average, the data point at each *x* (horizontal) position was obtained by averaging the bubble velocities along the vertical range of *y* = 0–120 mm (see Fig. 8). First, it is found that the time-averaged horizontal (Fig. 10a) and vertical (Fig. 10b) velocity components in general show the symmetric distribution with respect to the plume center at *x* ≈ 110 mm. The mean horizontal velocity represents the well-known diverging tendency of the bubble plume, which is negative (positive) at the left (right) side of the plume. It is noted that the pre-trained PWC-Net measures the horizontal velocity that is biased to the negative value, indicating the limitation in the multi-body identification. On the other hand, the fine-tuned CNN model is capable of capturing the stiff gradient (change) of the horizontal velocity across the center, another well-known feature of the bubble plume^52^, compared to other methods. From all the methods, the profiles of mean vertical velocity follow the bell shape, typically reported in the literature^22^. The results from the Farnebäck method and the fine-tuned PWC-Net are similar to each other, whereas the velocities obtained by the pre-trained PWC-Net are slightly lower than those from other algorithms. For the root-mean-squared velocity fluctuation (Fig. 10c,d), the results of the Farnebäck method and fine-tuned PWC-Net are higher than the others in both directions, since they are capable of reflecting the localized flow variations while the others make the velocity field smoothed, as discussed above. It is again noted that the Farnebäck method provided the highest root-mean-squared velocities, which is attributed to its sensitivity to the noise caused by the high-order assumption in intensity. Interestingly, depending on the methods, the r.m.s. velocity fluctuation is distributed in different manners; for example, the vertical component follows the concave (Lucas-Kanade method) or convex (others) profile (Fig. 10c). Currently, it is not clearly understood how this change is incurred and it would an interesting topic to investigate as a future work.

## Concluding remarks

In the present study, we have successfully demonstrated that the optical flow algorithms can be utilized as a new bubble velocimetry in experimentally investigating the gas–liquid two-phase flows in a very complex environment, such as the high-volume void fraction. As candidates for the potential algorithm, the conventional methods of Lucas-Kanade and Farnebäck model and CNN-based models of pre-trained and fine-tuned PWC-Net were tested using the high-speed bubble shadowgraph images obtained for three different configurations (single bubble rising near the solid wall, dilute bubble plume, and dense bubble plume). To optimize the optical flows, the effect of the interrogation window size (for the conventional optical flow) and the fine-tuning with the synthetic bubble images (for the CNN-based model) were systematically performed. Compared with the result of the PTV, it is found that the proposed optical flows, in general, correctly measure the temporal variation of the bubble velocity, in particular when there is only one bubble in the image. However, as the bubble number density increases substantially, which is our major motivation to develop new bubble velocimetry, the optical flow algorithms performed differently depending on their assumptions and level of training. For example, the CNN-based model that was not properly trained with the bubbly flow data did not perform well to capture the locally varying nature of bubble interface even in the case of dilute bubble plume, in which multiple bubbles simultaneously rise but rarely overlap. However, the re-trained CNN-based model (PWC-Net) was shown to be fully applicable even to the dense bubble plume, for which the traditional PTV approaches cannot produce any physically meaningful data (see the regions highlighted by dashed arrows in Fig. 8b). In overall, the Farnebäck and fine-tuned (re-trained) PWC-Net models tend to faithfully reflect the detailed spatial variation of each bubble velocity, being rigorously checked by comparing with the bubble motion from the raw image. Considering the accuracy of statistically higher-order flow variations and lower computational cost, on the other hand, it is concluded that the fine-tuned PWC-Net is recommended over the Farnebäck method as a new bubble velocimetry (see the supplementary move 3).

We have shown that the intensity-based (optical) algorithm can be very useful as an accurate measurement tool of bubble velocity, and hopefully replaces the traditional method (e.g., PTV), which uses the identification-based principle. To guarantee better accuracy and applicability, the physical relation between the interfacial deformation and the calculated velocity field inside the bubble should be elaborated, which may require detailed information and mechanism involved in bubble deformation (wobbling). This will be also useful to overcome the inherent limitation of the intensity-based algorithm such that it is hard to define the exact physical meaning of the evaluated velocity. Also, the change in the architecture of the CNN-based model should affect the performance significantly, which will be an interesting topic for future work.

**Figure 11:**
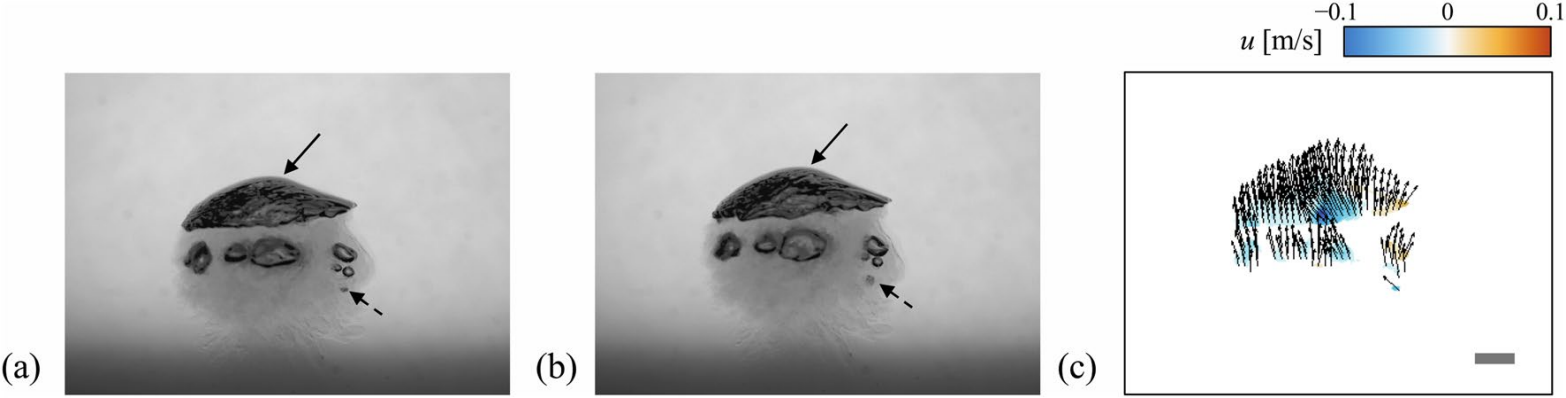
(**a,b**) Consecutive images (with a time separation of 2.5 ms) of the cap bubble and a few smaller bubbles. (**c**) Corresponding velocity field obtained by the fine-tuned CNN-based optical flow (the contour denotes the horizontal velocity). The solid bar in (**c**) denotes 10 mm.

While we have explained the possibility of the present velocimetry, it might be also worthy presenting the case in which the CNN-based optical flow fails to compute the velocity field correctly. Figure 11 shows the images of a rising cap bubble (with a lateral diameter of ~ 40 mm) and a few smaller bubbles (with a diameter of ~ 2 mm) around it, together with the entrained dye around the bubbles. Using the CNN-based optical flow (fine-tuned), the velocities of the large and small bubbles (denoted by the solid and dashed lines, respectively) are calculated (Fig. 11c). It is measured that the CNN-based optical flow provides the qualitatively reasonable velocity vectors according to the bubble motion: for example, the lateral motion of the small bubble is captured despite having a blurred edge due to the dye-induced light refraction. For the quantitative analysis, vertical (*u*) and horizontal (*v*) velocities of each bubble are calculated from the particle tracking velocimetry (PTV) and the CNN-based optical flow: for large bubble, (*u*, *v*) = (0.00 m/s, 0.28 m/s) with the PTV and (−0.02 m/s, 0.35 m/s) with the CNN-based optical flow; for small bubble, (*u*, *v*) = ( −0.22 m/s, 0.28 m/s) with the PTV and (−0.17 m/s, 0.18 m/s) with the CNN-based optical flow. Compared to the PTV result, thus the deviation of the CNN-based optical flow ranges 24–31%, which is not negligible. Especially, the largest error occurs for the small bubble, which suffers from the dye-induced refraction. This is quite large compared to the error (12.4%) for the dilute bubble plume (see Fig. 7). This is attributed to the light noise from the dye-induced refraction, which aggravates the vector calculation for all bubbles in the figure. This suggests that more rigorous preparation of the training data (for example, specified for the irregular shapes, existence wide range of scales, and noisy backgrounds) will be necessary to overcome this limitation.

Finally, we believe that the optimized CNN-model in the present study will also perform well for the other bubbly flows, since it was shown to successfully evaluate the two-phase flow (dense bubble plume) although it was not trained with the same data set. The optimized bubble velocimetry algorithm is available online (https://github.com/dae416/DeepBubbleVelocimetry), and we hope it can accelerate the further experimental investigations of the gas–liquid two-phase flows of a higher void fraction in a complex geometry.

## Methods

### The apparatus for bubbly flows and shadowgraphy

To establish the bubbly flows, the rectangular reservoir with the size of 1000 × 1000 × 1000 mm^3^ is filled with tap water and the air is injected through the sparger placed at the bottom of the reservoir, which is pressurized by the 4-HP air compressor (Airbank; AB350). The flow rate of air is controlled by the pressure regulator, through which the three different void fractions (*α*) are achieved: ~ 0 (single rising bubble), 1.13% (dilute bubble plume), and 58% (dense bubble plume) (Fig. 2a–c). With increasing *α* from 1.13 to 58%, the size distribution of bubble, which follows the typical log-normal curve^22^, broadens from 2.1 to 4.9 mm (where the median diameter, *d*_50_, is 3.25 mm) to 1.6–5.4 mm (*d*_50_ = 3.47 mm). Here, we used Sauter mean diameter for the size of deformable ellipsoidal bubble. For the dimensionless parameter, the Reynolds number ($Re={\rho }_{l}{d}_{50}{v}_{avg}{\mu }_{l}^{-1}$), Weber number ($We={\rho }_{l}{v}_{avg}^{2}{d}_{50}{\sigma }_{l}^{-1}$), and Froude number (*Fr* $={v}_{avg}^{2}/(g{d}_{50}^{2})$) ranges as 750–1,910, 2.4–15.0, and 510–2850, respectively. Here, ${\rho }_{l}$, ${\mu }_{l}$, and ${\sigma }_{l}$, denotes the density, dynamic viscosity, and surface tension of the liquid, respectively. And, *g* and ${v}_{avg}$ corresponds to the gravitational acceleration and the averaged vertical velocity of the bubble, respectively. The ratio of maximum and minimum size of the bubbles contained in the same image is around 29.5. To capture the bubble image, the light source and the diffusion plate are placed at one side of the reservoir, and the high-speed camera (Speedsense M310; Dantec Dynamics) is located at the opposite side, of which the spatial resolutions are 800 × 304 pixels (for the rising single bubble), 400 × 608 pixels (for dilute bubble plume), and 800 × 1280 pixels (for dense bubble plume) and the sampling rate is 1000 Hz (sufficient to capture the unsteady bubble shape and motion). Based on the convergence test, we have found that the bubble statistics are converged when they are averaged over 200 pairs of bubble images, which were adopted in this study to validate the measured bubble velocities.

### Conventional particle tracking velocimetry (PTV)

The PTV is performed using the in-house code, which consists of the binarization, identification, and evaluation, as shown in Fig. 1a–d. First, the shadow image of bubbles (or the bubble plume) is binarized using the Sauvola adaptive algorithm^7^. Then, the bright area inside the bubble is filled to avoid underestimating the bubble size. Next, the out-focused bubbles are excluded by thresholding the lower magnitude of intensity gradient at the bubble edge, and the overlapped bubbles are separated with the watershed transform^5^. For each time interval, the center locations of identified bubbles are collected. To evaluate the velocity vector, the bubble centers at two consecutive time instants are matched with the assumption that they are closest than others, while the outlier vectors are eliminated when the vector magnitude exceeds the prescribed threshold. Finally, the distance between location pairs is calculated and divided by the time interval between consecutive images, resulting in the bubble velocities (Fig. 1d). The procedure of the PTV is performed by CPU (Intel^®^ Core™ i7-5960X CPU @3.00 GHz), and the time costs for each sub-process are outlined in Table 1.

### Estimation of the uncertainty propagation

The uncertainty in the velocity measurement based on digital image acquisition comes from various hardware and software sources^53^. Since the velocity (*u*) is assumed to be a function of (*M*, *Δs*, *Δt*), the uncertainty is evaluated as $\delta (u)=\sqrt{\delta {\left(M\right)}^{2}+\delta {\left(\Delta \mathrm{s}\right)}^{2}+\delta {\left(\Delta t\right)}^{2}}$, based on the error propagation^5,8,53–55^. Here, *M* is the magnification factor, *Δs* is the object displacement during the time difference of *Δt*, and $\delta \left(*\right)$ is the relative uncertainty of the variable ‘*’. The magnification factor is determined by the calibration and its relative uncertainty was found to be approximately 0.7%. The relative uncertainty in the object displacement and the time separation of our setup was 1.8% and 0.05%, respectively. Finally, it is estimated that the uncertainty in measuring the velocity from the present optical figuration is around 1.93%.

### Architecture and detailed setting of PWC-Net (CNN)

**Figure 12:**
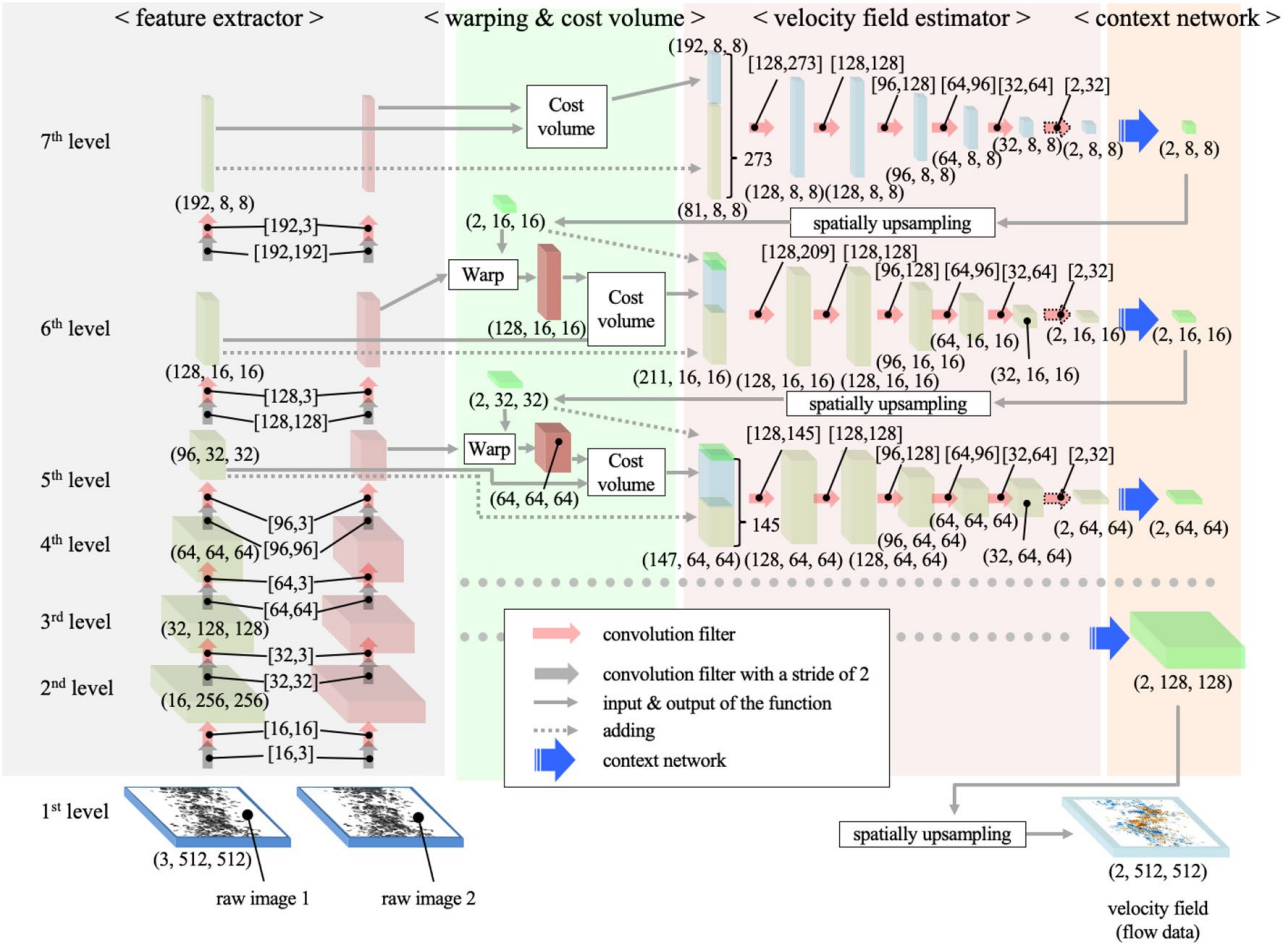
Architecture of the PWC-Net proposed by Sun et al.^35^.

Although some details about the tested CNN (i.e., PWC-Net), such as the number of layers, learning rate, and batch size were given in the main text, here we explain the detailed architecture and calculation procedure. Figure 12 shows the architecture of the PWC-Net proposed by Sun et al.^35^, which consists of the feature extractor, warping, cost volume, velocity field estimator, and context network. Based on this configuration, first, the two consecutive bubble images are fed into the same series of the convolution filters (i.e., Siamese network), which increases the depth of the feature (by increasing the number of the channel for the convolution filter) and coarsens its spatial resolution (by setting the stride as 2), resulting in 6 features for each bubble-image. At the 7th level (i.e. the lowest resolution), the correlation field (${c}^{7}$) is evaluated by the partial cost volume function, expressed as ${c}^{7}=1/N\cdot {\left({f}_{1}^{7}\right)}^{\mathrm{T}}{f}_{2}^{7}$, where *f* and *N* denote the feature and its length while the sub- and super-scripts correspond to the image order and the level of the feature, respectively. The correlation field padded with the ${f}_{1}^{7}$ is converted to the draft of the velocity field (${d}^{7}$) through the series of the convolution filters where the leaky ReLU is used as the activation function except for the last layer, which produces the ${d}^{7}$. And, as a post-processer, the context network finally generates the velocity field (${v}^{7}$) using the feed-forward CNN based on dilated convolutions to increase the receptive field size of the output^35,56^. Next, at the next-lower level (i.e. 6^th^ level), the two features (${f}_{\mathrm{1,2}}^{7}$) from the feature extraction are fed into the cost volume; however, in advance, the warping of the second feature, ${f}_{2}^{6}$, is implemented to compensate for the large motion and increase the precision, expressed as ${f}_{2w}^{6}={f}_{2}^{6}(x+2\cdot {v}_{up}^{7}(x))$, where *x* denotes the pixel location with fixed depth and ${v}_{up}^{7}$ is the formerly obtained velocity field which is up-sampled using the bilinear interpolation. And then the correlation field (${c}^{7}$) added with the first feature (${f}_{1}^{7}$) and the upscaled velocity field (${v}_{up}^{7}$) from the higher level is converted to the velocity field (${v}^{6}$) through the velocity field estimator and the context network (Fig. 12). This procedure is repeated until the velocity field (${v}^{3}$) at the third level is generated in this study. Since the spatial resolution is a quarter of the original image, ${v}^{3}$ is finally up-sampled by the bilinear interpolation to have the same resolution as the bubble image. Additionally, the training loss ($\mathcal{L}$) during the fine-tuning is computed by $\mathcal{L}\left(\Theta \right)={\sum }_{l=3}^{7}{\alpha }_{l}{\sum }_{x}\left({v}_{\Theta }^{l}\left(x\right)-{v}_{S}^{l}\left(x\right)\right)+\gamma {\left|\Theta \right|}_{2}^{2}$, where *x*, *l*, and $\Theta$, respectively, denote the pixel location, the feature level, and the set of every learnable parameter included in the feature extractor, velocity estimator, and context network. ${v}_{\Theta }^{l}$ and ${v}_{S}^{l}$, respectively, mean the predicted and supervised velocity field at the *l*^th^ feature level. The weights (${\alpha }_{l}$) for each level is set as 0.005, 0.01, 0.02, 0.08, and 0.32 for $l=$ 3, 4, 5, 6, and 7, respectively. ${\left|\cdot \right|}_{2}$ corresponds to the L1 norm, which regularizes networks’ parameters. The trade-off weight ($\gamma$) is selected as 0.0004.

## Supplementary Information

Supplementary Video 1.

Supplementary Video 2.

Supplementary Video 3.

## Data Availability

All data generated or analyzed during this study are included in this published article and supplementary information files.
